# Higher *Chlamydia trachomatis* Prevalence in Ethnic Minorities Does Not Always Reflect Higher Sexual Risk Behaviour

**DOI:** 10.1371/journal.pone.0067287

**Published:** 2013-06-14

**Authors:** Amy Matser, Nancy Luu, Ronald Geskus, Titia Heijman, Marlies Heiligenberg, Maaike van Veen, Maarten Schim van der Loeff

**Affiliations:** 1 Cluster of Infectious Diseases, Public Health Service of Amsterdam, The Netherlands; 2 Julius Centre for Health Sciences & Primary Care, University Medical Center Utrecht (UMCU), Utrecht, The Netherlands; 3 Department of Clinical Epidemiology, Biostatistics and Bioinformatics, Academic Medical Center, Amsterdam, The Netherlands; 4 Centre for Infection and Immunology Amsterdam (CINIMA), Academic Medical Center (AMC), Amsterdam, The Netherlands; Institut Jacques Monod - UMR 7592 CNRS - Université Paris Diderot, France

## Abstract

**Background:**

In affluent countries, the prevalence of Chlamydia trachomatis (CT) is often higher in certain ethnic minorities than in the majority population. In the Netherlands, we examined why CT prevalence is higher in Surinamese/Antilleans, the largest minority in the country.

**Methods:**

Heterosexuals were recruited for a cross-sectional survey from May through August 2010 at the sexually transmitted infections (STI) clinic in Amsterdam. Participants completed a questionnaire and were tested for STI. A causal directed acyclic graph was assumed to investigate whether the association between ethnicity and CT could be explained by differences in sexual risk behaviour and socio-economic status.

**Results:**

Subjects included 1044 with Dutch background and 335 with Surinamese/Antillean background. Median age for the combined population was 25 (IQR 22-30) years, and 55.4% was female. Sexual risk behaviour did not differ significantly between the two groups. CT was diagnosed in 17.9% of Surinamese/Antilleans and in 11.4% of Dutch. Surinamese/Antilleans were significantly more likely to have CT (OR 1.70; 95% CI 1.21-2.38). The association between ethnicity and CT remained statistically significant after adjusting for sexual risk behaviour, age, sex, and ethnic mixing (aOR 1.48; 95% CI 1.00-2.18), but not after adjusting for education and neighbourhood, markers of socio-economic status (aOR 1.08; 95% CI 0.71-1.64).

**Conclusion:**

The difference in CT prevalence between the minority and majority groups was not explained by differences in sexual risk behaviour. The higher CT prevalence found among Surinamese/Antilleans appeared to reflect their lower educational level and neighbourhood, two markers of lower socio-economic status. We hypothesise that the effect results from lower health-seeking behaviour.

## Introduction

In affluent countries, sexually transmitted infections (STIs) are usually more prevalent in certain ethnic minorities than in majority populations [1]. In the United Kingdom, the highest prevalence is seen among black Caribbean and African minorities [2,3]; in the United States, it is found in the African-American population [4,5]. In the Netherlands, the largest ethnic minorities are from Suriname or the former Dutch Antilles, Turkey, and Morocco. Among heterosexuals in the Netherlands, the most common STI is Chlamydia trachomatis (CT), which is most prevalent in the Surinamese/Antillean population. Their high CT prevalence has been found in several population-based studies and in data from STI clinics [6–8]. Whereas prevalence is around 11% in clinic attendees of Dutch background, it is close to 18% in those of Surinamese and Antillean background [9]. The challenge for policy makers and health care workers is to reach this high-risk population and reduce CT prevalence in the total population [6,10].

Typically, CT infection is primarily associated with sexual risk behaviours: unprotected sexual contact, multiple sex partners, young age, and young age at first intercourse [11]. Thus CT prevalence may differ among ethnic groups because of differences in sexual risk behaviour [3,12]. In some ethnic minorities, concurrent partnerships and inconsistent condom use are common [13–15] and might facilitate CT spread. Ethnic mixing patterns might be important as well. Most sexual partnerships are between individuals with the same ethnic background [14], and these assortatively mixed partnerships can assist STI spread within their subpopulation [4,16]. At the same time, disassortative or between-population mixing is common, and such partnerships might form a bridge for STI spread between populations [14,15].

The associations between CT and sexual risk behaviours are usually present in ethnic minorities but also in the majority population [17]. It is therefore unknown whether these determinants can actually explain a prevalence difference. In this study we try to explain why CT is more common among Surinamese/Antillean heterosexuals, compared to Dutch heterosexuals, by quantifying effects on the basis of a causal directed acyclic graph (DAG). As suggested by Fenton et al., different ethnic populations might need different prevention interventions that are targeted and culturally competent [3]. Unravelling the mechanisms that lead to differences in CT prevalence among ethnic populations might enable us to develop targeted intervention strategies adapted to the needs of diverse groups.

## Methods

### *Ethics statement*

Written informed consent was obtained from all participants. The study was approved by the medical ethics committee of the Academic Medical Center in Amsterdam.

### *Study population and setting*

The study population was recruited from the STI outpatient clinic of the Public Health Service of Amsterdam, the Netherlands. This clinic is the only STI outpatient clinic in the city and offers free and anonymous testing. Between May and August 2010, heterosexual clinic attendees were invited to participate in a cross-sectional survey until approximately 1000 men and 1000 women were included. To be eligible, they had to be at least 16 years old, visiting the clinic for a new consultation, and able to understand written Dutch or English. Men who had had sex with men in the preceding six months were excluded.

### *STI clinic procedures*

All attendees are routinely tested for urethral or vaginal CT and Neisseria gonorrhoeae (NG) infections, using NAAT (Aptima Combo, Gen-Probe, San Diego, CA USA). Depending on their sexual risk behaviour in the preceding 6 months, attendees are further tested for the presence of anal CT and NG infections and pharyngeal NG infections. HIV testing is performed unless an individual opts out [18], using a rapid immunoassay (Abbott Determine HIV 1/2; Abbott Diagnostic Division, Hoofddorp, The Netherlands), an HIV Ag/Ab Combo confirmation test (Axsym; Abbott Laboratories, Abbott Determine HIV 1/2; Abbott Diagnostics) and line-immunoassay (Inno-Lia HIV I/II Score; Innogenetics, Ghent, Belgium).

### *Covariates*

Participants completed an online questionnaire. In general, each question had to be answered before moving on to the next. However, questions could be skipped in a few cases where we judged that pressing for detail could cause confusion or resistance. The questionnaire addressed sociodemographic characteristics, sexual behaviour in general (eg, number of sex partners in lifetime and in the preceding year), specific sexual behaviour in self-defined steady or casual partnerships with up to four partners in the preceding year, and partners’ demographics. Demographic and clinical information on participants was also obtained from electronic patient records.

For this analysis, ethnicity was categorised as ‘Dutch’ if the participant and both parents were born in the Netherlands and as ‘Surinamese/Antillean’ if the participant or one of the parents was born in Suriname or the former Dutch Antilles. Education was categorised as lower (equivalent to elementary school, high school, or vocational training) and higher (equivalent to college or university). Information derived from the 4 digits of the Amsterdam postal code was used to determine the neighbourhood where the participant lived. 'The questionnaire contained multiple questions about sexual contact within up to 4 partnerships. Questions were asked per partnership. We asked whether the index had vaginal sex within the partnership in the preceding year. If yes, we asked how often they used condoms (options: never, seldom, sometimes, most of the times, or always). The same questions were asked for anal sex. We summarised the information of the 4 partnerships and categorised sexual contact as follows: (1) neither vaginal nor anal sex in the preceding year, (2) consistent condom use during vaginal and anal sex, (with all partners) and (3) inconsistent or absent condom use during vaginal or anal sex (with at least one partner).

Regarding each reported sexual partner, the questionnaire asked the month and year of first and last sexual contact. It did not ask for the exact day, as it seemed unlikely to be remembered accurately. To calculate partnership duration, we posited that partnerships started and ended on the 15^th^ day of the month, then subtracted the first date of sexual contact from the last date. The average duration of the reported partnerships per individual was calculated by summing the duration of all partnerships he/she reported and dividing the sum by the number of partnerships. Concurrency was categorised as (1) definitely non-concurrent, when there was at least one month between the described partnerships, (2) unknown, when the month of first sex contact in one partnership was the same as the month of last sex contact in another partnership, (3) and definitely concurrent, when at least one partnership overlapped more than one month with another partnership. Sex-related drug use was defined as recreational use of cocaine, 3,4-methylendioxymethamfetamine (XTC), γ-hydroxybutyric acid (GHB), amphetamines, sildefanil or other drugs shortly before or during sex with at least one partner in the preceding year.

### *Multiple imputations*

Before information per participant was summarised, missing values were imputed per partnership. Missing data as to partnership duration (n=927), ethnicity of the partner (n=8), condom use (n=25), and drug use (n=29) were imputed using multivariable imputation by chained equations [19]. The multivariable imputation prediction model included all variables of the final model plus the following: cohabitation, number of sex acts in the preceding year, number of lifetime partners, STI of the partner, and whether the partnership was ongoing. Twenty imputed data sets were created. Multiple imputations were done using the MICE package of R [19,20].

### *Statistical analysis*

**Figure 1:**
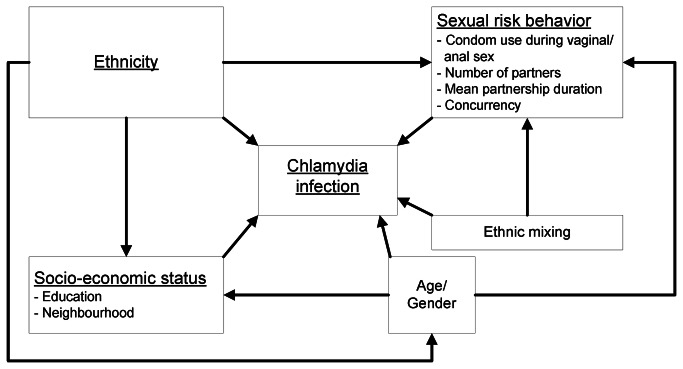
Causal directed acyclic graph. The direct and indirect pathways between ethnicity and chlamydia diagnosis are shown. Three main routes were identified through which ethnicity could be associated with chlamydia: (1) a direct biological route, (2) a direct route through sexual risk behaviour, and (3) an indirect route through socio-economic status. Age and gender were identified as possible confounders of the association between ethnicity and chlamydia, sexual risk behaviour and chlamydia, and socio-economic status and chlamydia. Ethnic mixing was assumed as a possible confounder of the association between sexual risk behaviour and chlamydia.

The main association studied was the association between ethnicity and CT diagnosis. We constructed a causal DAG in which to map the assumed pathways between ethnicity and CT diagnosis (Figure 1). We assumed three possible main routes, two direct and one indirect (ie, through other variables). The direct pathways are biological (ie, susceptibility) and sexual risk behaviour (eg, unprotected sex); the indirect pathway is mediated by socio-economic status. Age and gender were assumed to be possible confounders of the association between sexual risk behaviour and CT, between socio-economic status and CT, and between ethnicity and CT.

To capture sexual risk behaviour, we included the following covariates in multivariable analysis: condom use, number of partners in the preceding year, average duration of partnerships, and concurrency. Ethnic mixing was considered a possible confounder of the association between sexual risk behaviour and CT. We therefore distinguished participants with only assortatively mixed partnerships from those with only disassortatively mixed partnerships and those with both types.

Educational level and neighbourhood were also taken into account, because these two covariates are markers of socio-economic status [21,22]. We performed univariable and multivariable logistic regression analysis for every imputed dataset and pooled the results. Continuous variables (ie, age, number of partners in the preceding year, and average partnership duration) were modelled via restricted cubic splines with knots on the 2.5^th^, 25^th^, 50^th^, 75^th^, and 97.5^th^ percentiles. Data management was done using STATA Intercooled 11.2 (College Station, Texas, USA), and analysis was done using R [20].

## Results

During the study period, 5504 eligible individuals visited the STI clinic, of whom 2200 (40.0%) gave informed consent. No significant differences were found between the 2200 and the remainder in terms of gender, age, and number of sex partners in the preceding six months. To compare Dutch and Surinamese/Antillean individuals as to rate of participation (ie, giving consent), we used information about nationality (available on clinic records), as ethnicity was unknown for persons not responding to our questionnaire. There were 4528 individuals with a Dutch nationality and 77 individuals with a Surinamese/Antillean nationality. The percentages of individuals with a Surinamese/Antillean nationality did not differ significantly between those consenting (1.3%) and those not consenting (2.0%).

Based on responses to the questionnaire, individuals of other than Dutch or Surinamese/Antillean ethnic background were excluded from the current analysis (n=812); among these were 18 individuals with Moroccan ethnicity, 11 with Turkish ethnicity, and 29 of African ethnicity (these groups were too small for analysis). We also excluded individuals who did not report any partner or partnership characteristics (n=13). In total, 1375 individuals were included in the analysis: 1040 individuals had a Dutch ethnicity and 335 individuals had a Surinamese/Antillean ethnicity.

The median age of the 1375 individuals was 25 (IQR 22-30) years, and 55.4% of the participants was female (Table 1). A lower educational level was more common among Surinamese/Antilleans than among Dutch (63.6% vs. 23.5%; *P*<0.001). Mixing by ethnic background was common in both groups, with 51.8% of the combined population reporting at least one disassortative partnership. Concurrent partnerships were common in both groups. Dutch participants were more likely than Surinamese/Antilleans to have used drugs prior to or during sexual contact (18.2% vs. 6.6%; *P*<0.001). There were no significant differences regarding number of partners or consistent condom use. Over all, 11% were notified by a partner for a possible infection. For Surinamese/Antillean individuals, symptoms were more often the reason to visit the STI clinic: 42.7% versus 31.7% of the Dutch (*P*<0.001). CT was diagnosed in 17.9% of the Surinamese/Antilleans and in 11.4% of the Dutch (*P*=0.002) (Table 1).

**Table 1:** Characteristics, by ethnic background, of heterosexual individuals attending the sexually transmitted infections outpatient clinic, Amsterdam, the Netherlands, in 2010.

			Total population N=1375	Dutch participants N=1040	Surinamese/Antillean participants N=335	*P*-value
**Demographics**					
Female gender			761 (55.4%)	593 (57.0%)	168 (50.2%)	0.033
Median age in years (IQR)			25 (22-30)	25 (22-30)	25 (22-31)	0.643
**Socio-economic status**						
Education						<0.001
		Higher	918 (66.8%)	796 (76.5%)	122 (36.4%)	
		Lower	457 (33.2%)	244 (23.5%)	213 (63.6%)	
Neighbourhood						<0.001
		A (Centrum)	158 (11.5%)	144 (13.9%)	14 (4.2%)	
		B (Zuidoost)	124 (9.0%)	21 (2.0%)	103 (30.8%)	
		C (Oost-Watergraafsmeer)	107 (7.8%)	85 (8.2%)	22 (6.6%)	
		D (Oud-Zuid)	136 (9.9%)	130 (12.5%)	6 (1.8%)	
		Other in Amsterdam	540 (39.3%)	412 (39.7%)	128 (38.2%)	
		Outside Amsterdam	309 (22.5%)	247 (23.8%)	62 (18.5%)	
**Lifetime sexual behaviour**						
Median number of lifetime partners (IQR)			13 (6-21)	13 (7-21)	12 (5-25)	0.618
**Sexual behaviour in the preceding year**						
Median number of partners (IQR)			4 (2-6)	4 (2-6)	4 (2-6)	0.096
Consistent condom use			80 (5.8%)	56 (5.4%)	24 (7.3%)	0.305
Sex-related drug use with at least 1 partner^a^			211 (15.4%)	189 (18.2%)	22 (6.6%)	<0.001
Average partnership duration in days (IQR)			94 (19–307)	95 (24–297)	89 (1–379)	0.247
Concurrent partnerships						0.815
		No	414 (30.1%)	311 (29.9%)	103 (30.8%)	
		Unknown^b^	117 (8.5%)	91 (8.8%)	26 (7.8%)	
		Yes	844 (61.4%)	638 (61.4%)	206 (61.5%)	
Ethnic mixing						<0.001
		Assortative only	663 (48.2%)	559 (53.8%)	104 (31.0%)	
		Assortative & disassortative	451 (32.8%)	360 (34.6%)	91 (27.2%)	
		Disassortative only	261 (19.0%)	121 (11.6%)	140 (41.8%)	
**Sexually transmitted infection**						
Notified by partner as reason to visit the STI clinic			151 (11.0%)	110 (10.6%)	41 (12.2%)	0.456
Symptoms as reasons to visit the STI clinic			473 (34.4%)	330 (31.7%)	143 (42.7%)	<0.001
Chlamydia diagnosis			178 (13.0%)	118 (11.4%)	60 (17.9%)	0.002
Gonorrhoea diagnosis			32 (2.3%)	8 (0.8%)	24 (7.2%)	<0.001
HIV-infected			1	0	1	*

IQR = interquartile range; STI = sexually transmitted infection; HIV = human immunodeficiency virus;

a Recreational use of XTC, GHB, speed, cocaine, poppers, or sildefanil prior to or during sex;

b Concurrency could not be established when partnerships started in the same month as in which previous partnership ended.

In univariable analysis, the odds ratio (OR) for CT diagnosis was 1.70 (95% CI 1.21-2.39) for Surinamese/Antilleans compared to Dutch participants (Table 2). In multivariable analysis, we adjusted the association between ethnicity and CT for the covariates assumed in the DAG (Figure 1). The association remained statistically significant after adjusting for the possible confounding effects of age and gender. Sexual risk behaviour could not fully explain the association between ethnicity and CT, as the adjusted OR of Surinamese/Antillean ethnicity remained significantly increased after adjusting for number and duration of partnerships, consistent condom use during sexual contact in the preceding year, concurrency, and ethnic mixing (OR 1.48; 95% CI 1.00-2.18). The increased OR was best explained by low education and neighbourhood, which are markers of socio-economic status. After adjusting for these two markers and the possible confounders, the OR for Surinamese/Antilleans decreased to 1.08 (95% CI 0.71-1.64) and was no longer statistically significant.

**Table 2:** The association between chlamydia diagnosis and ethnicity estimated by logistic regression analysis among heterosexual participants attending the sexually transmitted infections outpatient clinic, Amsterdam, the Netherlands, in 2010.

		OR (95% CI)	*P*-value
Univariate		1.70 (1.21–2.39)	0.002
Adjusted for age and gender^a^		1.64 (1.16–2.31)	0.005
Adjusted for age, gender and sexual risk behaviour ^a,b,c^		1.48 (1.00–2.18)	0.050
Adjusted for age, gender, education, and neighbourhood^a^,^d^		1.08 (0.71–1.64)	0.730
Adjusted for age, gender, sexual risk behaviour, ethnic mixing, education and neighbourhood ^a,b,c,d^		0.97 (0.61–1.54)	0.910

OR = odds ratio; CI = confidence interval

a Continuous variables like age, number of partners in the preceding year, and average partnership duration were modelled as a restricted cubic spline with knots on the 2.5^th^, 25^th^, 50^th^, 75^th^, and 97 5^th^ percentiles;

^b^ Sexual risk behaviour included the following covariates: number of partners in the preceding year, average partnership duration, concurrency, inconsistent or absent condom use with a steady partner, inconsistent or absent condom use with a casual partner, and ethnic mixing;

^c^ Log transformations were used to model the number of partners in the preceding year and the average partnership duration;

d Education and neighbourhood were used as markers of socio-economic status.

## Discussion

Among visitors of the STI outpatient clinic in Amsterdam, the Netherlands, CT was more often diagnosed in those with Surinamese/Antillean background than those with Dutch background. Differences in age, gender and sexual behaviour between the ethnic groups could not explain the higher prevalence among Surinamese/Antilleans; however, it appeared to be explained by differences in education and neighbourhood, both markers for socio-economic status.

One of the strengths of this study, conducted at the largest STI clinic in the Netherlands, is the large number of participants who provided detailed information about their risk behaviour in the preceding year. This enabled us to correct for the most important covariates that might affect the association between CT and ethnicity, by adjusting for the covariates themselves, or by adjusting for proxies of the identified factors. We regard the study population as a good reflection of the clinic visitor population, as neither demographics nor STI prevalence differed between study participants and non-participants.

A study weakness was that recall bias might have affected our analysis, because questions were asked about behaviour in the year preceding participation and about details of partners and partnerships, that might have been forgotten. It is unknown how accurately participants reported their partners’ characteristics. Another study weakness was the amount of missing data, especially in variables measuring duration and timing of partnerships. The questionnaire was structured to avoid pressure that might tax the memory or the patience of respondees, evoking wild guesses or facetious answers. Regarding the first and last sex date with each reported partner, we asked only year and month, but not the day. Furthermore, questions on first and last date could be skipped, whereas all other questions had to be answered before moving on to the next. To minimize introduction of bias we used multiple imputation.

Extrapolation of our results to the general sexually active population must be performed with caution, because participants were recruited at an STI outpatient clinic, and their sexual risk behaviour might differ from the behaviour of the general population. The study captured 0.8% of the Dutch and 1.1% of the Surinamese/Antillean population in Amsterdam in the age-group 25-35 years.

In the DAG, we assumed three possible factors that could lead to a higher prevalence of CT in the Surinamese/Antillean population: higher sexual risk behaviour, a conducive biological mechanism, and lower socio-economic status. Differences in sexual risk behaviour could not fully explain the higher prevalence of CT among Surinamese/Antilleans, as ethnicity was still significantly associated with CT after adjusting for a range of relevant sexual risk behaviour variables. Because the association between CT and ethnicity became very weak in multivariable analysis, there is little evidence for a biological link between CT and ethnicity. Differences in education and neighbourhood could better explain the difference in CT prevalence. There was no longer statistical evidence of an association between ethnicity and CT after adjusting for educational level and neighbourhood.

This study suggests that the higher CT prevalence found among Surinamese/Antillean individuals in STI clinic-based studies is not caused only by higher sexual risk behaviour, but that it is mainly explained by low education and living in certain neighbourhoods, two markers of socio-economic status. Because health care insurance is obligatory for every Dutch resident and general practitioners are situated in every neighbourhood, health care access is comparable for all individuals and cannot explain why individuals of certain ethnic minority populations or in certain neighbourhoods are more often diagnosed with CT. We hypothesize that lower socio-economic status leads to an increased CT prevalence through lower health-seeking behaviour. In general, lower socio-economic status or low education can act as barriers to seeking medical help [23–25]. In some ethnic minorities, health-seeking behaviour might be further reduced by confidentiality issues and stigma [25–27]. The intertwined relationship between ethnicity and socio-economic status regarding CT infection has been described before, but the final conclusion about the importance of both covariates differed among the studies reviewed [11]. In a population-based CT screening project in the Netherlands, it was found that CT was associated with non-Dutch ethnicity and low socio-economic status [28]. It was also found that the screening rate was lower in subpopulations with higher CT prevalence [6].

Low health-seeking behaviour lowers the probability that a CT infection is detected and treated, thus leading to increased CT prevalence in the Surinamese/Antillean population. On average, Surinamese/Antilleans are infectious for a longer period of time than persons in the majority population. Individuals can therefore infect more susceptibles, not because they have more partners but because they have more time to do so. The prolonged infectious period in combination with assortative mixing (reported by more than 50% of the Surinamese/Antilleans) supports the already higher CT prevalence in this population. Since the two study populations were alike in number of partners per person, but the minority population is much smaller, its sexual network must be more closely connected.

Because recruitment occurred at a health facility, we could not adjust for health-seeking behaviour. We hypothesize that when health-seeking behaviour of a sub-population is low, its percentage of symptomatic clinic visitors will be high, as individuals are less likely to visit the clinic for routine screening. This is actually what we observed: 31.7% of Dutch and 42.7% of Surinamese/Antilleans visited the clinic because they had STI-related symptoms, suggesting that the Dutch population was more likely to visit the clinic for routine screening.

Mathematical models including ethnicity and population size of ethnic groups can provide further insight into the mechanism by which ethnicity is associated with CT. To date, several models have been developed to assess the impact of population-based CT screening [29,30]. Unfortunately, they did not account for differences in participation rates between majority and minority groups, even though they are known to be lower in sub-populations with high CT prevalence [6,24,28].

A second issue to examine is travel behaviour of migrants, especially on trips to their country of origin. Little is known about the prevalence of CT in Suriname or the former Dutch Antilles. In a study of women in Suriname, the CT prevalence ranged between 21% in an STI clinic and 9% in a more general medical setting [31]. This suggests that CT prevalence in the general population of Suriname exceeds prevalence in the majority population of the Netherlands. Approximately 60% of the Surinamese/Antilleans living in the Netherlands travel periodically to the country of origin, and 18.4% have a moderate or high risk to become infected abroad [32]. This influx of new infections combined with assortative mixing might contribute to an increased CT prevalence.

Further research in this and other ethnic groups is necessary, as mechanisms may differ among various groups. This hypothesis is supported by findings in the United Kingdom. The prevalence of risky sexual behaviour among black and white ethnic groups in the UK was explained by age, sex, and marital status. There were no differences in risky sexual behaviour between black and white ethnic groups after accounting for the explanatory variables. Among Indians and Pakistani, the prevalence of risky behaviour was lower than what could have been expected based on age, sex, and marital status [33]. In the Netherlands, for example, the CT prevalence in Turkish/Moroccan migrants is lower than that of the majority population, but their socio-economic status is no higher than that of the Surinamese/Antilleans. Among Turkish/Moroccans, lower sexual risk behaviour or different mixing patterns, instead of socio-economic status, might explain the difference in CT prevalence. For the development of effective prevention strategies, it is important to consider the mechanisms that explain CT prevalence in different ethnic groups, because different mechanisms need different strategies. More research is needed to increase our understanding of the mechanisms other than just sexual risk behaviour that lead to increased STI prevalence in certain ethnic minorities. Mixing patterns in and between neighbourhoods might be important, as is ethnic mixing, and also the distance to clinics might play a role. Along with the existing strategies, it might be effective to develop strategies on neighbourhood level and to conduct opportunistic screening through local general practitioners in certain neighbourhoods.

To conclude, we found that the prevalence among Surinamese/Antilleans is best explained by education and neighbourhood, two markers of socio-economic status, and might be due to lower health-seeking behaviour. We found no evidence for an independent association between ethnicity and CT diagnosis and no differences in sexual risk behaviour between Dutch and Surinamese/Antillean individuals that can explain the difference in CT prevalence.
